# 

**DOI:** 10.3325/cmj.2014.55.78a

**Published:** 2014-02

**Authors:** Gregor Nosan, Sara Bertok, Samo Vesel, Helger G. Yntema, Darja Paro-Panjan

In Reply: We agree that “antimongoloid palpebral slant” is a pejorative term, although it has been used by some contemporary authors for describing different syndromes (1-4). When a word enters our everyday practice it is sometimes difficult to replace it with a new, more technical and appropriate term. We are grateful for the comment (5) and wholeheartedly encourage the use of proper terminology, especially in scientific articles.
